# Using Predictive Analytics to Identify Children at High Risk of Defaulting From a Routine Immunization Program: Feasibility Study

**DOI:** 10.2196/publichealth.9681

**Published:** 2018-09-04

**Authors:** Subhash Chandir, Danya Arif Siddiqi, Owais Ahmed Hussain, Tahira Niazi, Mubarak Taighoon Shah, Vijay Kumar Dharma, Ali Habib, Aamir Javed Khan

**Affiliations:** ^1^ Harvard Medical School Center for Global Health Delivery–Dubai Dubai Healthcare City United Arab Emirates; ^2^ Interactive Research and Development Baltimore, MD United States; ^3^ Interactive Research and Development Karachi Pakistan; ^4^ Interactive Health Solutions Karachi Pakistan

**Keywords:** machine learning, artificial intelligence, immunizations, dropouts, predictive analytics

## Abstract

**Background:**

Despite the availability of free routine immunizations in low- and middle-income countries, many children are not completely vaccinated, vaccinated late for age, or drop out from the course of the immunization schedule. Without the technology to model and visualize risk of large datasets, vaccinators and policy makers are unable to identify target groups and individuals at high risk of dropping out; thus default rates remain high, preventing universal immunization coverage. Predictive analytics algorithm leverages artificial intelligence and uses statistical modeling, machine learning, and multidimensional data mining to accurately identify children who are most likely to delay or miss their follow-up immunization visits.

**Objective:**

This study aimed to conduct feasibility testing and validation of a predictive analytics algorithm to identify the children who are likely to default on subsequent immunization visits for any vaccine included in the routine immunization schedule.

**Methods:**

The algorithm was developed using 47,554 longitudinal immunization records, which were classified into the training and validation cohorts. Four machine learning models (random forest; recursive partitioning; support vector machines, SVMs; and C-forest) were used to generate the algorithm that predicts the likelihood of each child defaulting from the follow-up immunization visit. The following variables were used in the models as predictors of defaulting: gender of the child, language spoken at the child’s house, place of residence of the child (town or city), enrollment vaccine, timeliness of vaccination, enrolling staff (vaccinator or others), date of birth (accurate or estimated), and age group of the child. The models were encapsulated in the predictive engine, which identified the most appropriate method to use in a given case. Each of the models was assessed in terms of accuracy, precision (positive predictive value), sensitivity, specificity and negative predictive value, and area under the curve (AUC).

**Results:**

Out of 11,889 cases in the validation dataset, the random forest model correctly predicted 8994 cases, yielding 94.9% sensitivity and 54.9% specificity. The C-forest model, SVMs, and recursive partitioning models improved prediction by achieving 352, 376, and 389 correctly predicted cases, respectively, above the predictions made by the random forest model. All models had a C-statistic of 0.750 or above, whereas the highest statistic (AUC 0.791, 95% CI 0.784-0.798) was observed in the recursive partitioning algorithm.

**Conclusions:**

This feasibility study demonstrates that predictive analytics can accurately identify children who are at a higher risk for defaulting on follow-up immunization visits. Correct identification of potential defaulters opens a window for evidence-based targeted interventions in resource limited settings to achieve optimal immunization coverage and timeliness.

## Introduction

Despite the availability of free routine immunizations in low- and middle-income countries (LMICs), many children are not completely vaccinated, are vaccinated late for age, or drop out from the course of the immunization schedule. According to the World Health Organization (WHO) and United Nations International Children's Emergency Fund (UNICEF) immunization coverage estimates, the mean dropout rates for Bacillus Calmette–Guérin (BCG) and the second dose of measles-containing vaccine are 34.6% (SD 20.4%) in low-income countries and 28.6% (SD 20.4%) in GAVI-eligible LMICs [1]. Studies have reported consistent findings in which the coverage rates of earlier vaccines are significantly higher than the coverage for vaccines that are administered later on in the immunization schedule, [2,3] with the highest dropout occurring between the diphtheria-tetanus-pertussis (DTP3) dose and the first dose of measles vaccine [2]. A probable explanation is the relatively long time interval (35.5 weeks) between the administration of the DTP3 vaccine (14 weeks) and measles vaccine (9 months), which increases the likelihood of mothers forgetting about the vaccination appointment or not having the time to make scheduled visits for immunizations [3].

Despite individual efforts by governments to improve coverage and reduce dropout rates, vaccinators lack readily available on-site information tools to target children who are at highest risk of dropout or late vaccination. To achieve full universal coverage and improve the timeliness of individual vaccine doses, low-resource countries can model and visualize the risk on large datasets, including that at the individual level during immunization visits, to identify and target children who are at a high risk of dropping out or delaying the next vaccine dose.

In the era of big data, when the collection of massive amounts of reliable data has become inexpensive and easy, predictive analytics is being utilized in a wide variety of settings. The fields of business, marketing, and finance were among the earliest adopters of predictive analytics. One well-known application is credit scoring, a predictive model that analyzes a particular customer’s information, such as credit history, to assess the potential risk of lending money to that customer. Web-based retailers, such as Amazon, also utilize powerful predictive algorithms to tailor item recommendations for the individual experience of their users [4].

Predictive analytics technology uses mathematical and computational statistical modeling, machine learning, and multidimensional data mining techniques [5] to accurately forecast future immunization outcomes based on existing data and to predict parental adherence to routine childhood immunization schedules. What makes predictive analytics powerful and so widely applicable is the fact that the systems can iteratively learn and improve over time [5] to achieve the desired quality of predictive performance. These systems use traditional statistical methods, such as the calculation of the area under the system’s receiver operating characteristic (ROC) curve, to measure the system’s predictive performance [6]. It was not until electronic medical records and big data in health care became more widely adopted that opportunities for using predictive analytics in health began to increase [7]. A machine learning algorithm built to optimize the management of patients with chronic kidney disease in the United States was able to identify the most probable data-driven clinical pathway and predict the upcoming required intervention with an accuracy of 50%-75% [8]. A proof-of-concept study at the Department of Medicine at Yale University created a random forest model and “trained” it to predict the in-hospital mortality rate of patients with sepsis. The model used local data from the hospital, and it had an area under the curve (AUC) with a 95% CI of 0.86 (range 0.82-0.90), outperforming all traditional analytic models used as controls with statistically significant results [9]. In addition to anticipating outcomes based on the population level, predictive analytics have also been used to forecast individual outcomes. Researchers at the University of Texas, Houston, developed three machine learning algorithms to predict suicidality among individuals with mood disorders based on their medical and sociodemographic data. All three models had >50% accuracy in distinguishing someone as an individual who had attempted to commit suicide from someone who had not [10].

According to WHO, in 2015 [11], a child born in a low-income country was 11 times more likely to die before reaching the age of 5 years than a child born in a high-income country, highlighting the crucial link between demographic and socioeconomic factors influencing health outcomes. Our hypothesis is as follows: a child’s likelihood to miss or not show up on time for a vaccination visit is correlated with certain demographic and background characteristics, such as socioeconomic status, gender, maternal education, ethnicity, and location. We have leveraged the power of “big data” collected through a digital immunization registry to develop a predictive analytics algorithm that tags children who are most likely to miss their follow-up immunization visits. Through statistical modeling, we can use immunization and demographic data to classify whether a child showing up at the immunization center is at high or low risk of missing subsequent immunization visits. This research aimed to develop and validate the accuracy of the predictive analytics algorithm in identifying children who were likely to default from subsequent immunization visits for any vaccine included in the routine immunization schedule. We also sought to determine which predictive analytics model has the highest predictive accuracy. Although our research was based on previous studies about behavioral predictive analytics models, this will be the first to examine parental adherence to routine childhood immunization schedules in developing countries.

## Methods

### Study Population and Data Source

Vaccination data were abstracted from the Zindagi Mehfooz Digital Immunization Registry, a mobile phone-based registry program initially supported by the United Nations Foundation and currently scaled in Sindh province with support from WHO. The registry software was developed based on an android platform, and it has various features, including web interface, mobile phone-based data access and entry, radio frequency identification and quick response code-based identification, interactive short message service (SMS) reminders, electronic decision support system that guides vaccinators for routine and catch-up immunizations, and geographic information system for tracking of vaccinators. The retrospective data subset had 49,439 records from 21 immunization centers in two cities (Karachi, Sindh and Muzaffargarh, Punjab) collected from May 2012 to April 2016. We excluded a total of 1885 records from the total dataset; among these, 326 records were excluded based on invalid dates for age or immunizations and three were not included because the children had died. Moreover, 1556 were excluded because they only had measles-2 immunization record, which is the last recommended immunization dose, and there were no further follow-up visits.

**Figure 1:**
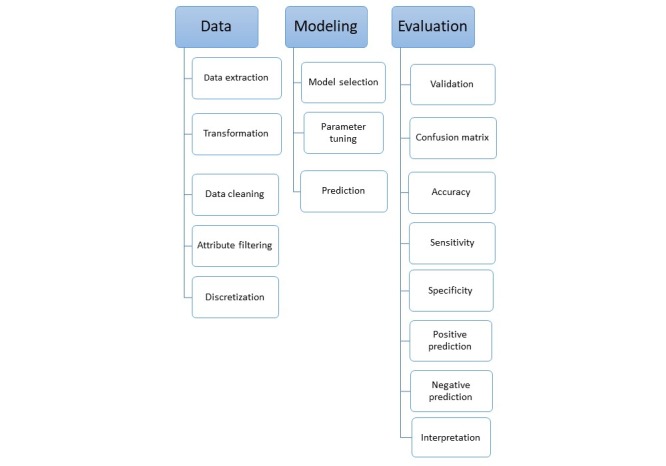
Procedures of the Study.

The cohort of children included in the model had visited the immunization center for one of the six routine immunization visits. These children had complete records of the core variables used in the analysis. During data extraction, transformation, and cleaning stage, the information on demographic and vaccine-related variables was obtained as raw data. The variables for model prediction were used from routinely collected data on the Expanded Program on Immunization (EPI) for administering recommended immunizations to children aged below 2 years. The variables that did not add any contextual information (child’s name, address, and contact number) were filtered out, whereas the rest were utilized in the model (Textbox 1). Figure 1 summarizes the main procedures of the study.

### Data Analysis or Prediction Objective

Our primary objective was to validate the functionality of the predictive analytics model through predicting the likelihood of each child defaulting from subsequent immunization visits for any vaccine included in the routine immunization schedule.

### Modeling

We used support for recursive partitioning, support vector machines (SVMs), random forests, and C-forest models in the predictive analytics component. These models were encapsulated in the predictive engine, which identified the most appropriate method to use in a given case based on the following standard measures: accuracy, precision (positive predictive value), sensitivity, specificity, and negative predictive value.

#### Recursive Partitioning

Recursive partitioning is a statistical method that creates a binary decision tree that classifies the classes of the target attribute by recursively splitting the training data into subsets until a certain criterion is met. The advantage of recursive partitioning algorithm is its performance on larger datasets and flexibility in prioritizing sensitivity and specificity. However, the disadvantages include overfitting data and the lack of support for continuous variables. Furthermore, the problem of overfitting can be resolved with the use of tuning parameters [12].

#### Support Vector Machines

SVMs are based on a discriminative classification technique that forms a tree-like graph of learned classification rules. This model is extremely efficient for binomial target attributes, and it performs well on datasets with a high number of attributes, regardless of training data size. This study uses LibSVM implementation [13,14].

#### Random Forests

Random forests are an extension of the decision tree model. The random forest grows several trees against each classification rule, each providing a classification of a target object. The decision is made through voting. The benefit of using random forests is their higher accuracy on larger datasets and their capability to handle high-dimensional data without the need of using the dimensionality reduction step. Random forests are also good at locating outliers and scaling data to reduce error due to bias. Breiman’s implementation [15,16] of the random forest has been used in this study.

**List of predictors from the routinely collected immunization data**

1. Gender of the child
2. Language spoken at the child’s house
3. Place of residence of the child (town or city)
4. Enrollment vaccine
5. Timeliness of vaccination
6. Enrolling staff (vaccinator or others)
7. Date of birth (accurate or estimated)
8. Age group of the child (<1 month, 1 month, 2 months, 3 months, 4 months, 6 months, 9 months, 1 year, 1.5 years, 2 years, 3 years, and >3 years)

#### C-Forest

C-Forest is based on conditional inference trees, which estimate a regression relationship by binary recursive partitioning in a conditional inference framework. C-Forest can work on multivariate target variables as well, which is not supported by the recursive partitioning model by default. This study used an algorithm proposed by Hothorn, Hornik [17].

### Parameter Tuning

In this step, the default parameters of the algorithms were tuned on different values until the most optimal setting, for example, the values of the parameters that provide the best accuracy for the model, had been reached. These parameters were different for each algorithm; for example, in the random forest model, we discovered that the default value for the number of trees to grow (50) was insufficient. Thus, we tested different values and chose 150 as the optimal value. Another example from the recursive partitioning is complexity parameter in which we determined the algorithm if the complexity parameter was set to 0.01; then, a node should had split further only when the goodness of fit was improved to at least 0.01 due to this split. We learned that the default value (0.01) was appropriate and changing it did not improve the results.

For parameter tuning, the training dataset was further split into two parts: training set and validation set. Classifiers were trained on training set and tuned upon the test set. Then, the final accuracy was measured on the validation set in which the outcome of the target variable was hidden from the classification algorithm. Although parameter tuning could improve accuracy (often extremely marginal), this was an optional step.

### Evaluation

For evaluating the algorithm, we carried out bootstrapping to generate training and validation dataset. To avoid affecting the performance of the model, the validation dataset was not included as part of the training set. The validation dataset was generated as follows:

1. Extracting a sample of size equal to the dataset with replacement
2. Storing all observations from the dataset for validation, which were not selected during sampling
3. Repeating the sampling until the size of the validation set is one-fourth (11,889) of the original dataset size (47,554).

**Figure 2:**
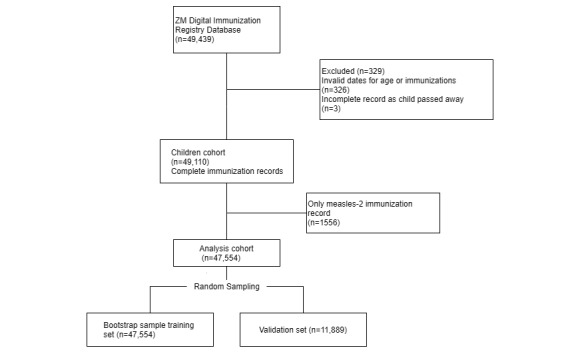
Derivation procedure for extracting training and validation cohort data. ZM: Zindagi Mehfooz.

This validation dataset set was neither used during training nor for parameter tuning. It was only used for model evaluation. Random sampling with replacement from the original sample was performed until the training subsample equivalent to the same size as the original sample was achieved. All the left-over records, which were not selected in the training set, were placed together in the validation subsample, as seen in Figure 2. The test set was separated initially, and no parameter tuning was performed on this set to ensure the simulation of real-world data population. These test data were later used to test the accuracy of the other parameters of each model by predicting the target class.

Accuracy, which is defined as the percentage of total correct predictions, is considered the first parameter in the evaluation of any machine learning algorithm: accuracy = (T_P_ + T_N_) / (T_P_ + T_N_ + F_P_ + F_N_), where T_P_ refers to all correct positive classifications, T_N_ indicates all correct negative classifications, F_P_ represents all false positive classifications, and F_N_ refers to all false negative classifications. The other parameters included the following: sensitivity = T_P_ / (T_P_ + F_N_), specificity = T_N_ / (T_N_ + F_P_), precision (positive predictive value) = T_P_ / (T_P_ + F_P_), and negative predictive value = T_N_ / (T_N_ + F_N_). The rationale behind using multiple parameters is that accuracy is not the de facto model in every case; for example, in the case of predicting immunization, we might prefer an algorithm with high sensitivity over another algorithm with higher accuracy. Furthermore, the overall prediction accuracy of all machine learning models was measured using the area under the ROC curve (C-statistic). ROC curve is a plot of true positive rate [T_P_ / (T_P_ + F_N_)] against the false positive rate [F_P_ / (F_P_ + T_N_)], and AUC determines the predictive performance of the model.

## Results

The baseline characteristics of the children in the test and validation cohorts are shown in Table 1. Both subsets had similar characteristics in terms of the selected variables. The mean enrollment age was 12.9 weeks, and the highest enrollment was carried out during the BCG vaccination visit. The baseline demographic characteristics of the participants excluded from the analysis (n=256) were not significantly different from those included in the final analysis (N=47,554). Out of 11,889 cases in the validation dataset, the actual number of children who defaulted was 6155.

**Figure 3:**
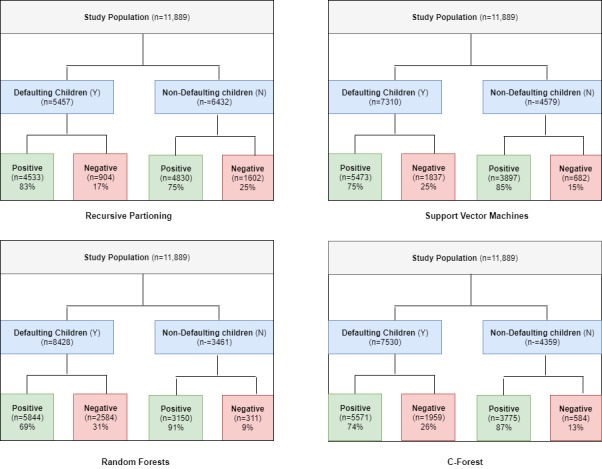
Flow diagram of all the study predictive models.

Figure 3 provides a visual illustration of the outcomes of all models showing the number of true positives, true negatives, false positives, and false negatives.

According to the four outcomes produced, the recursive partitioning model predicted that 45.90% (5457/11,889) children would default; among them, 83.43% (4553/5457) children did default, which accounts for 83.4% of the total default population. Likewise, it was predicted that 54.10% (6432/11,889) children would return for the next vaccination; among them, 75.09% (4830/6432) children did return. In the support vector machine model, the total population of children who defaulted was 7310 (7310/11,889, 61.48%); among them, 5473 defaulted, which accounts for 74.87% (5473/7310) of the total default population. Likewise, it predicted that 38.51% (4579/11,889) children would return for vaccination; among them, 85.11% (3897/4579) did return. Meanwhile, the random forest model predicted that the total number of children who defaulted will be 70.89% (8428/11,889); among them, 69.34% (5844/8428) did default. Likewise, it predicted that 29.11% (3461/11,889) children would return for vaccination; among them, 91.01% (3150/3461) did return. Lastly, the C-forest model predicted that 63.34% (7530/11,889) would default; among them, 73.98% (5571/7530) did default. Likewise, it predicted that 36.66% (4359/11,889) children would return for vaccination; among them, 86.20% (3775/4359) did return. These results produced accuracy rates of approximately 78.9%, 78.8%, 75.6%, and 78.6% for recursive partitioning, SVMs, random forests, and C-forest, respectively (Table 2).

**Table 1:** Baseline characteristics of the training and validation data cohorts.

Characteristics of the participants					Training cohort (N=47,554)		Validation cohort (N=11,889)
Enrollment age (weeks), mean (SD)				12.92 (15.9)		12.93 (15.9)	
Gender (female), n (%)				20,425 (42.95)		5049 (42.47)	
**Enrollment vaccine, n (%)**							
	BCG^a^			24,744 (52.03)		6195 (52.11)	
	Pentavalent-1			8955 (18.83)		2236 (18.81)	
	Others			13,855 (29.14)		3458 (29.08)	
**Language spoken, n (%)**							
	Urdu			846 (1.78)		208 (1.75)	
	Unknown			46,561 (97.91)		11,644 (97.94)	
	Others			147 (0.31)		37 (0.31)	
**Place of residence (town), n (%)**							
	Korangi			41,225 (86.69)		10,296 (86.60)	
	Muzafargarh Town			1693 (3.56)		445 (3.74)	
	Others			4636 (9.75)		1148 (9.66)	
**Place of residence (city), n (%)**							
	Karachi			45,415 (95.50)		11,334 (95.33)	
	Muzafargarh			1996 (4.20)		519 (4.37)	
	Others			43 (0.30)		36 (0.30)	
**Timeliness of vaccination^b^, n (%)**							
	**BCG**						
		Early		16 (0.07)		4 (0.07)	
		Late		17,126 (70.19)		4254 (69.61)	
		Timely		7258 (29.75)		1852 (30.32)	
	**Pentavalent-I**						
		Early		11 (0.12)		1 (0.02)	
		Late		8892 (99.73)		2220 (99.78)	
		Timely		13 (0.15)		4 (0.18)	
	**Pentavalent-II**						
		Early		9 (0.22)		2 (0.20)	
		Late		4099 (99.15)		996 (99.20)	
		Timely		26 (0.63)		6 (0.60)	
	**Pentavalent-III**						
		Early		14 (0.38)		3 (0.34)	
		Late		4338 (99.31)		883 (99.21)	
		Timely		11 (0.30)		4 (0.45)	
	**Measles-I**						
		Early		6 (0.14)		1 (0.09)	
		Late		4338 (99.20)		1113 (99.02)	
		Timely		29 (0.66)		10 (0.89)	
	**Age group**						
		<1 month		5465 (11.49)		1386 (11.66)	
		1-9 months		35,972 (75.64)		8949 (75.27)	
		>1 year		6117 (12.86)		1554 (13.07)	

^a^BCG: Bacillus Calmette–Guérin.

^b^Excludes records with invalid dates.

**Table 2:** Performance of the study models predicting the likelihood of defaulting from the follow-up immunization visits. Higher C-statistics results in better algorithm discrimination*.*

Model	Area under the curve C-statistic	95% CI
Recursive partitioning	0.791	0.784-0.798
Support vector machines	0.786	0.777-0.792
Random forests	0.750	0.742-0.756
C-Forest	0.782	0.775-0.789

Overtime, through using artificial intelligence (AI), because more data are captured, the system will continue to self-learn from accumulated records, recognizing influential variables, self-selecting statistical models, and continually upgrading itself to achieve the highest predictive accuracy. However, the recursive partitioning model outperforms the rest of he models in terms of overall accuracy rates, but since the performance of a classifier does not directly depend on the accuracy rate alone, therefore, we analyzed other performance metrics, such as sensitivity, specificity, positive predictive value, and negative predictive value. Table 3 presents the outcomes for all the performance metrics.

**Figure 4:**
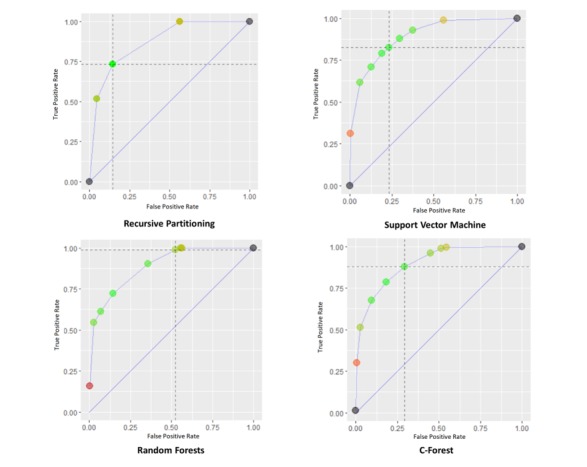
Receiver operating characteristic for all the study predictive models.

According to Table 3, the random forest model outperforms all the other models with a sensitivity rate of 94.9%, although it has the lowest accuracy rate. The random forest model predicted that majority of the population will default, that is, it has predicted that (70.88% of the whole population, 8428/11,889) will default. Moreover, it can correctly identify the maximum number of children who defaulted (5844 out of 8428 children actually defaulted). The random forest model’s high sensitivity permits the recognition of almost all children who will not receive subsequent vaccinations (94.9%). By contrast, the recursive partitioning model produces the highest specificity at 84.2% and lowest sensitivity at 74.0%, indicating that it can identify the maximum number of children who will adhere to their vaccination schedule. The recursive partitioning model produces moderate results for both sensitivity and specificity at 74.0% and 84.2%, respectively, and it had the highest accuracy rate at 78.9%. Figure 4 shows the individual performance metrics for each model as illustrated in the ROC.

The random forest model correctly predicted 8994 cases, yielding a sensitivity and specificity of 94.9% and 54.9%, respectively. The C-forest model, SVMs, and recursive partitioning models improved the prediction by achieving 352, 376, and 389, additional correct cases, respectively, over the predictions made using the random forest model. However, looking across the models, as accuracy of the models increased, the sensitivity decreased from 94.9% (for random forest model) to 74.0% (for recursive partitioning model), whereas specificity went up from 54.9% (for random forest model) to 84.2% (for recursive partitioning models). All models had a C-statistic of 0.750 or above, and the recursive partitioning model algorithm had the highest statistic (AUC 0.791, 95% CI 0.784-0.798; Table 2).

**Table 3:** Performance metrics of all the study predictive models.

Model	Accuracy (%)	Sensitivity (%)	Specificity (%)	Precision (%)	Negative predicted value (%)
Recursive partitioning	78.9	74.0	84.2	83.4	75.1
Support vector machines	78.8	88.9	68.0	74.9	85.1
Random forests	75.6	94.9	54.9	69.3	91.0
C-Forest	78.6	90.5	65.8	74.0	86.6

## Discussion

### Principal Findings

We have demonstrated the feasibility and validity of the predictive analytics algorithm in identifying children who were likely to default from subsequent immunization visits, and the algorithm yielded a 79.1% accuracy rate. This information could empower policy makers, immunization programs, and vaccinators to reduce dropouts and improve immunization coverage, timeliness, and equity through the targeted use of evidenced-based interventions at an individual or community level. Reduced immunization coverage and losses to follow-up do not allow communities to fully take advantage of the benefit of routine childhood immunization programs.

Because the approach is becoming a topic of interest, results from initial formative studies on the use of predictive analytics in a variety of settings are now being assessed. Our findings are in accordance with those reported from other studies that have used AI technology within the health domain to predict future outcomes. The success rates of predictions from other studies are similar; for example, a model conducting risk profiling of patients who are likely to develop chronic kidney disease using gradient tree-based algorithm had an AUC statistic of 0.871, and statistically significant (*P*<.001) differences were observed in disease outcomes in the high-, medium-, and low-risk groups [18]. Similarly, in another study that predicted cardiovascular risk, the predictions produced by the machine learning algorithm using a variety of models were better (AUC 0.745, 95% CI 0.739-0.750) than those produced by the existing risk prediction algorithms [19]. These findings corroborate the potential of predictive analytics to revolutionize the current practices of preventing disease and promoting better health care.

This formative study tested the feasibility of an array of statistical models to make predictions showing the variability of results depending upon our outcome of interest. The random forest model had the best performance with results expected to further improve as more data is collected because the system learns overtime as a result of machine learning. Other studies that have used different predictive models also reinforce the finding that one of the models is typically the highest achieving model compared with others depending on the outcome of interest [19]. The selection of variables for the predictive model was limited to the information collected during routine immunizations. Machine learning will also proactively interpret and identify new data patterns in routinely collected data, significantly improving the accuracy of individual risk classification over time. However, collecting additional variables, including household income, ethnicity, maternal tetanus vaccination status, and maternal and paternal education status, may further enhance the predictive accuracy.

Operationally, developing countries are in the process of using digital immunization registries (DIRs), which provide an extremely rich source of patient information [20], creating an opportunity for effectively using machine learning and predictive analytics to identify children who are most likely to default from their immunization schedule. From a technical standpoint, predictive analytics has high interoperability, which helps it to be easily linked to any DIR or electronic health record to strengthen the health systems and empower the vaccinators. This feature further enhances the utility of this module given the high appeal for interoperability to enable cooperative progress in public health through linking heterogeneous data [21].

To further enhance the ease of use, the front end of the module is designed for nonprogrammers, and it does not require technologically skilled users, making it easy to implement and sustain in low-resource settings. From an operational perspective, the utilization of predictive analytics does not require large investments in resources or trainings. With the expanding presence of DIRs, the technological platform for large-scale implementation is already in place, and the user interface can be tailored to meet local requirements. The self-learning algorithm quickly adapts to context, adjusting variables, models, and standard measures as needed.

Financially, the returns to be gained from optimal resource allocation and reduced expenditure on vaccine-preventable diseases are substantially greater than the set-up cost, ensuring a high return on investment per dollar spent. Although a high-dropout may mean that a large proportion of the population must be targeted at the start, the offset in the required funding may be substantial for LMICs. Other clinical studies that used AI for predicting future outcomes also highlight the reduction in economic burden through early detection and treatment of disease [22]. The health department and local government could ultimately benefit through savings incurred owing to the allocation of resources to population segments that require them the most. Wasting of the limited resources of the government could be reduced if not eliminated. Furthermore, the health department could make substantial savings in the treatment costs for vaccine-preventable diseases.

In addition, machine learning techniques have also been proven to improve resource allocation decisions. For instance, a study examining patient admission decisions in tertiary care hospitals has revealed that a machine learning Bayesian model could lead to more efficient resource allocation decisions when deciding which patients to admit in the hospital. Similarly, in our context, predictive analytics can identify children at high risk for overburdened frontline health workers and as a result, evidence-based interventions, such as center-based counseling, out-reach services, and repeated SMS reminders, can be targeted toward this cohort leading to optimal resource allocation.

Our idea constitutes an unconventional approach for improving the timeliness of routine immunization and reducing missed opportunities; in an era where a collection of massive amounts of reliable data has become cheap and easy, predictive analytics is considered a cutting-edge innovation with only limited application in the field of health service delivery despite its strong impact and potential. Machine learning, particularly deep learning, is now being used to predict the patients’ chances of relapse, early deterioration, and developing diseases, such as cancer and automated diagnosis of eye disease, as recently shown by Google. However, in the field of immunization, predictive modeling is a novel idea, and its potential in revolutionizing immunization service delivery is yet to be identified.

To achieve the key goal of the global vaccine action plan 2011-2020, for example, meet the 90% national vaccination coverage and 80% coverage rate for all vaccines by 2020 in every district, we need to focus on strategies that reduce dropouts and expand coverage. As presented in this paper, predictive analytics can help in the identification of children who are likely to default or dropout from the course of the immunization schedule; therefore, communities where incomplete immunization rates are prevalent will benefit the most from targeted concentration of efforts promoting the goal of universal health equity. Although this paper provides a plausible causal pathway in which the information gained through this model can lead to health system improvement, more rigorous evaluations must be conducted to fully determine the programmatic effectiveness of this model from an implementation perspective.

### Limitations

The limitation of our model was the exclusion of the records containing invalid dates for age or immunizations. Although the imputation method was used to deal with invalid or missing data in the machine learning models because this was a feasibility study, the data models were utilized only on complete records. Furthermore, it is relevant to mention that we have evaluated the predictive analytics algorithm on only one outcome, particularly the likelihood of a child to default from subsequent immunization visits. There are other parameters in which the algorithm could be evaluated, such as the likelihood of completing the full immunization schedule. However, to keep the approach simple, other approaches were considered beyond the scope of this study, and this must be further evaluated. The predictive analytics will be beneficial for communities with high access and underutilized services because the model is based on initial contact with vaccinator or health care worker, and communities with low access may only benefit indirectly when herd immunity is achieved. The other limitation of the study is the generalizability of data to other populations. Developing this model for other populations would require recalibration and adjustment to account for other disparities as well as the inclusion of relevant prediction variables.

### Conclusion

The expansion of DIRs in lower- and middle-income countries is creating a unique opportunity to analyze and interpret data to generate real-time actionable insight in expanding immunization services and coverage. This feasibility study showed that predictive analytics can accurately identify individual children who are likely to default from subsequent immunization visits. Predictive analytics can strengthen immunization programs by facilitating the targeted implementation of interventions aimed at reducing the dropouts.

## Abbreviations

AI: artificial intelligence
AUC: area under the curve
BCG: Bacillus Calmette–Guérin
DIR: digital immunization registries
DTP3: diphtheria-tetanus-pertussis
EPI: Expanded Program on Immunization
FN: false negative
IRD: Interactive Research and Development
LMIC: low- and middle-income countries
ROC: receiving operating characteristic
SMS: short message service
SVM: support vector machines
TP: true position
UNICEF: United Nations International Children's Emergency Fund
WHO: World Health Organization

## References

[1] (2017). World Health Organization.

[2] Sadoh AE, Eregie CO (2009). Timeliness and completion rate of immunization among Nigerian children attending a clinic-based immunization service. J Health Popul Nutr.

[3] Onyiriuka A (2009). Vaccination default rates among children attending a static immunization clinic in Benin City, Nigeria. Vol.

[4] Linden G, Smith B, York J (2003). Amazon.com recommendations: item-to-item collaborative filtering. IEEE Internet Comput.

[5] Cody S (2014). and A. Asher, Smarter, Better, Faster: The Potential for Predictive Analytics and Rapid-Cycle Evaluation to Improve Program Development and Outcomes, Mathematica Policy Research.

[6] Linden A, Yarnold Paul R (2016). Using data mining techniques to characterize participation in observational studies. J Eval Clin Pract.

[7] Bates DW, Saria S, Ohno-Machado L, Shah A, Escobar G (2014). Big data in health care: using analytics to identify and manage high-risk and high-cost patients. Health Aff (Millwood).

[8] Zhang Yiye, Padman Rema (2015). Innovations in chronic care delivery using data-driven clinical pathways. Am J Manag Care.

[9] Taylor R, Pare Joseph R, Venkatesh Arjun K, Mowafi Hani, Melnick Edward R, Fleischman William, Hall M Kennedy (2016). Prediction of In-hospital Mortality in Emergency Department Patients With Sepsis: A Local Big Data-Driven, Machine Learning Approach. Acad Emerg Med.

[10] Passos I, Mwangi Benson, Cao Bo, Hamilton Jane E, Wu Mon-Ju, Zhang Xiang Yang, Zunta-Soares Giovana B, Quevedo Joao, Kauer-Sant'Anna Marcia, Kapczinski Flávio, Soares Jair C (2016). Identifying a clinical signature of suicidality among patients with mood disorders: A pilot study using a machine learning approach. J Affect Disord.

[11] (2017). World Health Organization.

[12] Friedman J (1977). A Recursive Partitioning Decision Rule for Nonparametric Classification. IEEE Trans. Comput.

[13] Cortes Corinna, Vapnik Vladimir (1995). Support-vector networks. Support-vector networks.

[14] Chih-Chung CC (2011). -J. L., LIBSVM: A library for support vector machines. ACM Transactions on Intelligent Systems and Technology (TIST).

[15] Breiman L (2002). , Manual on setting up, using,understanding random forests v3. 1, Statistics Department University of California Berkeley, CA, USA.

[16] Breiman L (2001). Random Forests. Machine Learning.

[17] Hothorn T (2006). , K. Hornik, and A. Zeileis, Unbiased Recursive Partitioning: A Conditional Inference Framework. Journal of Computational and Graphical Statistics.

[18] Hao S, Fu T, Wu Q, Jin B, Zhu C, Hu Z, Guo Y, Zhang Y, Yu Y, Fouts T, Ng P, Culver DS, Alfreds ST, Stearns F, Sylvester KG, Widen E, McElhinney DB, Ling XB (2017). Estimating One-Year Risk of Incident Chronic Kidney Disease: Retrospective Development and Validation Study Using Electronic Medical Record Data From the State of Maine. JMIR Med Inform.

[19] Weng SF, Reps J, Kai J, Garibaldi JM, Qureshi N (2017). Can machine-learning improve cardiovascular risk prediction using routine clinical data?. PLoS One.

[20] Daniele RC (2017). W., Fani Deligianni, Deep Learning for Health Informatics. IEEE Journal of Biomedical and Health Informatics.

[21] Jaulent M, Assélé-Kama A, Savard S, Giavarini A, Touzé E, Jeunemaître X, Ugon A, Plouin P, Toubiana L (2015). Building a Semantic Interoperability Framework for Care and Research in Fibromuscular Dysplasia. Stud Health Technol Inform.

[22] Danner OK, Hendren S, Santiago E, Nye B, Abraham P (2017). Physiologically-based, predictive analytics using the heart-rate-to-Systolic-Ratio significantly improves the timeliness and accuracy of sepsis prediction compared to SIRS. Am J Surg.

